# Experience and Perceptions of Retention Strategies in District Nursing Services: A Web‐Based Mixed Methods Cross‐Sectional Survey

**DOI:** 10.1111/jan.70108

**Published:** 2025-08-01

**Authors:** Erkan Alkan, Vari M. Drennan, Claire Thurgate, Lihua Wu, Mary Halter, Chao Wang

**Affiliations:** ^1^ Faculty of Health, Science, Social Care & Education Kingston University London UK; ^2^ Institute of Health, Education & Science Kingston University London UK; ^3^ School of Nursing, Allied and Public Health Kingston University London UK

**Keywords:** community health, cross sectional studies, health workforce, nurses, nursing services, working conditions

## Abstract

**Aim:**

To investigate the experience and perceptions of the effectiveness of retention strategies of nurses and nursing associates in district nursing services.

**Design:**

Mixed methods cross‐sectional online survey.

**Methods:**

Electronic invitations were circulated via district nursing professional networks to complete an online survey in England. The survey questions were developed from international evidence‐based guidance. Quantitative data were analysed descriptively and using multinomial regression analysis, tested the variation in experienced strategies by job and work characteristics. Content analysis informed qualitative data analysis.

**Results:**

Three hundred and forty‐five completed surveys were received. Over 60% of **r**espondents reported experiencing strategies related to a safe working environment (75%), flexible work schedules (65%), well‐being (64%) and professional development opportunities (60%). The least frequently reported strategies experienced were involvement in service policymaking (26%), reducing job demands (31%); and creating cohesive nursing teams (40%). Nurses on lower pay grades were statistically less likely than those on the higher pay bands to experience strategies involving professional growth opportunities and involvement in service decision‐making. Nurses working in affluent areas were statistically more likely to report experiencing more types of retention strategies than those working in socio‐economically deprived areas. Participants' views on effective strategies were mixed but attention to financial aspects (particularly travel costs), manageable workloads, flexibility in work scheduling plus tailored induction/support for those new to district nursing were given the most testimony as effective.

**Conclusions:**

Retention strategies are created and enacted by those within employing organisations, district nursing services and district nursing teams, but within the context of a wider health care and labour market system. We suggest the findings could be the starting point for review by district nursing services experiencing high vacancy rates. Our findings raise questions for subsequent investigation across health systems.

**Patient Reporting Method:**

This paper adhered to the relevant Equator guideline A Consensus‐Based Checklist for Reporting of Survey Studies (CROSS), https://doi.org/10.1007/s11606‐021‐06737‐1.

**Patient or Public Involvement:**

This study did not include patient or public involvement in its design, conduct or reporting.

**Summary**

- What problem did the study address?
  ○ Retaining nurses in district nursing services is a problem in many countries with such home visiting nursing services. The work environment of home visiting nurses is very different from that of hospital nurses, on which evidence for retention of nurses is derived.
- What were the main findings?
  ○ This web‐based cross‐sectional survey found wide variation in experience and perceptions of retention strategies in nurses in district nursing services.
  ○ Nurses working in affluent areas were statistically significantly more likely to report experiencing more types of retention strategies than those working in socio‐economically deprived areas.
  ○ Views on effective strategies were mixed but attention to financial aspects (particularly concerned with travel costs), manageable workloads, flexibility in work scheduling, plus tailored induction/support for those new to district nursing were given the most testimony as effective.
- Where and on whom will the research have an impact?
  ○ The research offers new information for nurses, clinical managers and policy makers for reviewing retention strategies for district nursing services, with particular consideration of equitable application to all members of individual teams and across regions.
- What does this paper contribute to the wider global clinical community?
  ○ This paper provides new information on retention strategies for those countries with existing home visiting nursing services or considering the establishment of such services, for example, hospital at home services.

## 1 Introduction

Internationally, health systems are looking to ways to meet the needs of their changing populations, which have increased prevalence of chronic illnesses, within their economic and financial constraints. One policy solution has been to increase the provision of treatment and care in community settings and in people's homes (World Health Organization 2023). Nurses, skilled in providing care in the home, are required to support such a policy shift. Many, but not all, countries have such nursing services; variously known as home healthcare nursing, visiting nursing and district nursing (the term used in this paper) (Drennan 2019). Countries such as the United States (US) and Sweden have reported severe shortages of nurses in district nursing services since the global pandemic (Gaines 2021; SCB 2020). The World Health Organization (WHO) and the International Council for Nurses (ICN) have argued that all health systems and employers should pay attention to retention strategies for nurses (World Health Organization 2016; Buchan et al. 2022). It is not evident to what extent nurses in district nursing services experience these advised retention strategies, or to what extent the nurses view them as effective for district nursing services. This paper reports on a study that investigated these issues.

## 2 Background

There is no internationally agreed definition of district nursing. In those countries with such services, the majority of patients are aged over 65 with multiple co‐morbidities and usually mobility issues (see, for example evidence, from the Netherlands [Veldhuizen et al. 2021], Japan [Fukui et al. 2014], US [CDC National Centre for Health Statistics 2024]). The nursing services typically provide long‐term care, including in acute episodes of illness, as well as palliative and end‐of‐life care. Many countries are seeking to increase health care delivered at home rather than in resource‐intensive hospitals, such as through more acute hospital‐atat‐home services (see, for example, Canada [Canadian Journal of Health Technologies 2024], the United Kingdom, UK [Schiff et al. 2022]) and terminal care at home (see, for example, Norway [Ervik et al. 2023]). All of these types of developments require a nursing workforce skilled at delivering care in peoples' homes. Nursing in peoples' homes is a very different work environment to that of health centres or hospitals, where colleagues are close at hand and there are no physical travel or connectivity problems to the internet or phone signals (Drennan, Thurgate, et al. 2025). Across the world, there are known shortages and maldistribution of nurses (Sharplin et al. 2025). Similarly, there have been shortages reported of registered nurses (RNs) to work in district nursing services in many countries such as Canada (Ohashi et al. 2024), Japan (Ottawa Citizen 2021) and Sweden (SCB 2020). The staffing in district nursing services is typically organised into teams which include RNs, practical nurses (known in some countries as licensed practical nurses LPNs or registered practical nurses RPNs) and nursing assistants. High levels of retention of nurses maintain service quality and continuity, while reducing overhead costs of recruitment and reliance on temporary staffing to cover vacancies (World Health Organization 2016; Buchan et al. 2022). Rates of nurses leaving an organisation are one measure of nurse retention. A 2023 Canadian study reported higher average leaver rates 2016–2020 in home care services compared to hospital services for RNs (24% in home care and 14% in hospitals) and RPNs (64% in home care and 19% in hospitals) (Drost and Sweetman 2023). A US home health nursing organisation reported the average annual leaver rate of RNs and LPNs between 2016 and 2019 was over 30%, across its 200 local home health agencies or branches in 30 states (Bergman et al. 2022). The method of calculating the average was not specified, but this does suggest higher and lower figures for different branches of the same organisation. Evidence from England reports annual turnover rates for RNs to vary from 5% to 40% between different district nursing service organisations (NHS Benchmarking 2023), indicating variation between employing organisations. One possible explanation for this observed variation in retention rates could be differential use of retention strategies between different organisations.

The WHO stated that health professional retention strategies should provide decent working conditions in all settings and elaborated this as including the following: fair salary and terms of employment; occupational health and safety; merit‐based opportunities for career development; a positive work environment to ensure motivation in providing quality health care; appropriate levels of autonomy and involvement in organisational decision making (World Health Organization 2016). The State of the World's Nursing 2020 report used findings from systematic reviews to argue that retention strategies specific for nurses should include attention to addressing gender pay gaps (in recognition that most nurses are female); flexibility in work schedules; supportive supervisory line management; manageable workloads and clear career pathways (World Health Organization 2020). The ICN, using their updated previously published review of nurse retention evidence, added the strategy of paying attention to nurses' health and well‐being because of the evidence from the COVID‐19 pandemic (Buchan et al. 2022).

While there are some studies examining the job satisfaction and intention to leave of nurses in district nursing services (Senek et al. 2023; Kaihlanen et al. 2023) there are surprisingly few that examine the use and impact of retention strategies. A recent scoping review of evidence since 2000 (Drennan, Ferrer, et al. 2025) identified a 2001 survey of home health agency administrators' views which favourably reported the effects of decreasing job demands and increasing financial rewards and benefits (Cushman et al. 2001). The review found one other study from 2007 that analysed US nurses' self‐reported intention to stay in the context of their employing home care agency's administrators' self‐reported retention strategies (Ellenbecker et al. 2007). This study reported that none of the individual retention strategies directly affected the nurses' intention to stay but also noted that the extent to which the retention strategies were implemented was unknown (Ellenbecker et al. 2007). The voice of nurses, who work in patient‐facing roles, as to their experience and views of the effectiveness of retention strategies, in the context of different employing organisations was absent from the literature (Drennan, Ferrer, et al. 2025). This paper reports on research which investigated this evidence gap in order to inform nurse managers and policy makers in their efforts to address the problem of retention rates of nurses in district nursing services.

The aim of this study was to investigate the perceptions of nurses and nursing associates in district nursing services as to what types of retention strategies they had experienced, whether that experience varied by job and work characteristics such as their seniority and whether they perceived the retention strategies as effective. The research questions of the study were:

1. Did nurses and nursing associates in the district nursing services in England experience all the retention strategies recommended by international guidance for retaining nurses?
2. Did the nurses and nursing associates in the district nursing services experience of retention strategies vary by: job seniority; type of employing organisations; the urban, rural or socio‐economic status of the work area?
3. Which of the retention strategies recommended by international guidance did the nurses and nursing associates perceive as effective?

## 3 Methods

### 3.1 Study Design

An online anonymous mixed methods cross‐sectional survey was used to investigate the perceived exposure to retention strategies and views of effectiveness of those strategies from nurses and nursing associates in district nursing services in England. An online survey method was chosen as a low‐cost method with wide geographical reach. The use of mixed methods, that is, structured questions and free text, was chosen to explore the research questions more broadly and identify topics for further research (Garcia et al. 2004). The recommendations of the Consensus‐Based Checklist for Reporting of Survey Studies (CROSS) (Sharma et al. 2021) have been followed in reporting this study.

### 3.2 Setting

The study was undertaken in England. England is one of the four countries of the UK which has district nursing services throughout. The district nursing services are paid for by the National Health Service (NHS), a tax‐funded healthcare system which is free to patients at the point of care. The NHS offers national terms and conditions for nurses, supported by workforce policies and a suite of human resource management resources including retention strategies (NHS Employers 2024). The NHS has a national pay structure, known as pay bands, with defined salary ranges. Pay bands are a numbered system in which registered nurses (RNs) commence at pay band 5 and the band number rises for jobs with additional clinical and/or managerial responsibilities (NHS Employers 2024). Nursing associates' jobs (a new role in the UK and described as a bridge between health care assistants and RNs) are classified as pay band 4 (NHS Employers 2024). In 2024 district nursing services in England were commissioned and paid for by 42 local NHS integrated care boards and provided by 76 different organisations (NHS Digital Care Contacts 2024). Most of the provider organisations were NHS organisations (known as trusts) but some of these only provided community services while others also provided hospital or mental health service NHS trusts. A small number of the 76 provider organisations were not‐for‐profit and for‐profit organisations outside of the NHS (NHS Digital Care Contacts 2024). District nursing services variously employed registered nurses with additional specialist practitioner qualifications, known as district nurses (DNs), registered nurses (RNs), nursing associates (NAs) and health care assistants. In 2024 there were 29,841 full‐time equivalent RNs and NAs employed in district nursing services in England (3,866 DNs, 25,000 RNs, 975 NAs) (NHS England Digital 2024).

### 3.3 Participants, Recruitment and Procedures

The study aimed to recruit nurses and nursing associates in district nursing services in every region of England, employed at different pay grades and by different types of organisations; and working in areas of different socio‐economic status as well as urban and rural areas. The inclusion criteria were (a) a registered nurse (including those with district nurse qualifications) or nursing associate and (b) employed in district nursing services in England. The exclusion criteria were working (a) in health services or organisations other than district nursing services; (b) in health care assistants' positions; or (c) in countries other than England.

Participants were recruited via five national professional online networks for nurses in district and community services. Each of these networks used social media, on‐line message boards or electronic newsletters to communicate with their members. In May 2024 the networks posted or circulated the invitations, which had been prepared by the research team, to participate in the survey. The invitation outlined the study's purpose, instructions to complete the anonymous survey, the estimated time to complete it, consent and ethical issues, data protection measures and the details about the research team members. The invitation provided the link to the online survey, where the participant information was repeated. Reminder invitations were circulated via these networks once in June 2024. None of the networks provided membership numbers at the time of the invitation.

In order to incentivise completion of the survey, participants could choose to join a separate lottery for one of three shopping vouchers (value £20, Great Britain pounds, equivalent to $26 US dollars). They could also choose to receive the report and publications from the research. Two online links, separate from the survey and therefore protecting anonymity in their survey responses, were provided to leave contact details for entry to the lottery and to receive updates on the study.

### 3.4 The Survey Instrument and Measurements

As there was no existing survey instrument relevant to the research questions, one was developed by the research team. Face and content validity of the survey was established through meetings and discussions with the study expert advisory group. This group comprised 15 DNs, RNs and senior nurse administrators of district nursing professional organisations and services from across England. The expert advisory group also recommended the survey be brief in order to increase the likelihood of completion.

The research team first identified the types of retention strategies for nurses recommended in international guidance (World Health Organization 2016; Buchan et al. 2022). Eleven types of strategies were identified: professional growth opportunities; flexible work schedules; control over their own work activities; financial benefits; creating cohesive nursing teams; employee recognition; reducing job demands; ensuring a safe working environment; active involvement in the development of the policies of the organisation; promotion of staff well‐being; and strategies to address the needs of specific subgroups such as internationally educated nurses. Each of the 11 strategies was used in a question in the survey instrument as to whether the participant had experienced that retention strategy. Examples of each strategy were given as relevant to district nursing services as suggested by an independent advisory group to the research study team. Answer options were a drop‐down list of ‘yes’, ‘no’ or ‘maybe’. Participants were then invited to provide a free text response as to whether they considered that strategy effective or not.

The survey was piloted with five registered nurses and nursing associates working in district nursing services, independent of the research team. The pilot participants were asked to provide feedback about whether the questions made sense, and if not, which ones needed rewording; questions that they considered should be added; how to improve the survey and make it more likely that nurses and nursing associates would complete it; and the length of time it took them to complete. The median time for completion was 15 min and the suggestions of the pilot participants were incorporated into a reworded second version of the survey.

The amended survey was then used to create an online survey using Microsoft Forms (Data S1). The structured survey had questions on whether the participant had experienced, or not, each of the 11 retention strategies, followed by free text options for their views of the effectiveness of these strategies. Data were collected on the respondent's job title; NHS national pay band; job environment (type of working area); NHS region of England; and employing organisation type.

Respondents were able to review their responses before submission, but once submitted, they could not resubmit. Microsoft Forms automatically assigned a unique three‐digit number to each participant. Data was collected from May to July 2024.

### 3.5 Ethical Considerations

Full information about the purpose and conduct of the study was given in the invitation to participate. Participants were informed that their consent was implied by completing the survey and no separate consent process was required. No personal information was collected in the survey responses that could identify participants. Participants could choose to participate in the separate lottery and/or receive study updates by providing their contact details on separate Microsoft Forms not connected to their survey responses. The study (application 3338) was reviewed and approved by a University Research Ethics Committee.

### 3.6 Data Analysis

The anonymous data were extracted from the Microsoft Forms platform and stored on the password‐protected university drive. Quantitative and qualitative analyses were undertaken, initially by two researchers and then discussed and reviewed with the wider research team.

#### 3.6.1 Descriptive Statistics

Descriptive statistics were calculated to summarise the work characteristics of the participants, including NHS pay band, geographic settings of the working area, socio‐economic characteristics of the working area, region of the working area and type of employing organisation. Frequencies and percentages were used to describe the distribution of participants across these categories. Similarly, descriptive statistics were used to present the number and percentage of participants who responded ‘Yes’, ‘No’, or ‘Maybe’ to each survey item.

#### 3.6.2 Multinominal Logistic Regression Analyses

To answer research question 2, we ran a multinomial logistic regression analysis, testing the variation in experienced strategies by job and work characteristics. Separate models were estimated for each outcome variable (i.e., strategy experienced) containing the three possible responses: ‘Yes’, ‘No’ and ‘Maybe’, with ‘No’ as a reference category. The measures were:

- Independent variables: NHS pay band categories (NHS pay band 4 to 8); work area by socio‐economic categories (mixed with pockets of affluence and deprivation, mainly socio‐economically affluent, deprived); type of employing organisation (NHS community service trust, community interest company, NHS community and mental health service trust, and NHS acute and community service trust); type of work area by geographic settings (town, inner city, rural and mixed urban/rural).
- Outcome variables: The outcomes of interest were the likelihood of responding ‘Yes’, ‘No’ and ‘Maybe’ to experiencing each type of retention strategy. We treated both maybe and yes responses as having experienced the strategy.

Each model included all independent variables to estimate their effects on the likelihood of each response category relative to the reference category (‘No’). Coefficients, standard errors, *z*‐values, *p*‐values and 95% confidence intervals were calculated for each independent variable. The following subgroups were omitted due to the multicollinearity: mainly socio‐economically affluent work area, NHS community health and mental health trust, and mainly rural work area.

The statistical significance of independent variables was assessed using *p*‐values, with significance levels set at *p* < 0.05. Model fit was evaluated using the likelihood‐ratio index and pseudo‐*R* ^2^ values (Menard 2000). All analyses were conducted using STATA 14 software.

#### 3.6.3 Free Text Analysis

Free text responses were exported to Microsoft Excel spreadsheets. Content analysis was undertaken by two researchers (Spencer et al. 2014). Both researchers had prior experience of research investigating the retention of health professionals. The content analysis included quantitative and qualitative analysis. The frequency of comments about each strategy was recorded first, and the strategies were ranked by volume of comments. The qualitative element of analysis was initially inductive as each free‐text response was specific about a type of retention strategy. The multiple responses to each type of retention strategy were read and re‐read, initially grouping responses into two themes as to whether they were perceived as effective or ineffective. The researchers read and then deductively assigned codes, which were subthemes regarding any evidence or explanations for the participants' views. Disagreements between the researchers were resolved through discussion. The findings were reviewed and discussed with the wider team.

## 4 Results

### 4.1 Characteristics of Participants

Three hundred and fifty‐two unique individuals completed the survey and seven were excluded from the district: five for not meeting inclusion criteria of working in district nursing services; and two for starting the survey but not completing it. The results were based on answers from 345 participants. The descriptive statistics of the participants' characteristics are given in Table 1. The largest group of participants was in jobs in NHS pay band 5 (108 participants, 31.3%) followed by Band 6 (81 participants, 23.5%) and Band 7 (82 participants, 23.8%). The highest percentage of participants worked in mixed urban and rural areas (115 participants, 33.3%), followed by town areas (92 participants, 26.7%). Most participants worked in areas that were socio‐economically mixed with pockets of affluence and deprivation (241 participants, 69.9%). The participants worked in all NHS England regions, with the Northwest region and Northeast and Yorkshire region having the highest representation (68 participants for each, 19.6%). The largest group of participants worked in NHS community service trusts (148 participants, 43.2%), followed by NHS acute and community service trusts (115 participants, 33.6%).

**TABLE 1:** Descriptive statistics of participants' characteristics.

Characteristics		
NHS pay band	*N* = 345	%
4	22	6.4
5	108	31.3
6	81	23.5
7	82	23.8
8a	30	8.7
8b	14	4.1
8c	4	1.12
8d	2	0.6
9	2	0.6
Geographical setting of work area	*N* = 345	%
Mixed urban and rural	115	33.3
Town	92	26.7
Mainly rural	67	19.4
Outer city and/or suburbs	50	14.5
Inner city	21	6.1
Socio‐economic characteristics of the work area	*N* = 345	%
Mixed with pockets of affluence and deprivation	241	69.9
Mainly socio‐economically deprived	65	18.8
Mainly socio‐economically affluent	39	11.3
NHS region of work area	*N* = 345	%
Northwest	68	19.7
Northeast and Yorkshire	68	19.7
Midlands	55	15.9
Southeast	49	14.2
Southwest	48	13.9
East of England	37	10.7
London	20	5.8
Type of organisation	*N* = 342	%
NHS community service trust	148	43.2
NHS acute and community service trust	115	33.6
NHS community and mental health service trust	46	13.5
Community interest company (or other social enterprise business)	29	8.5
Limited company (or other type of for‐profit business)	4	1.2

### 4.2 Retention Strategies Experienced

**FIGURE 1:**
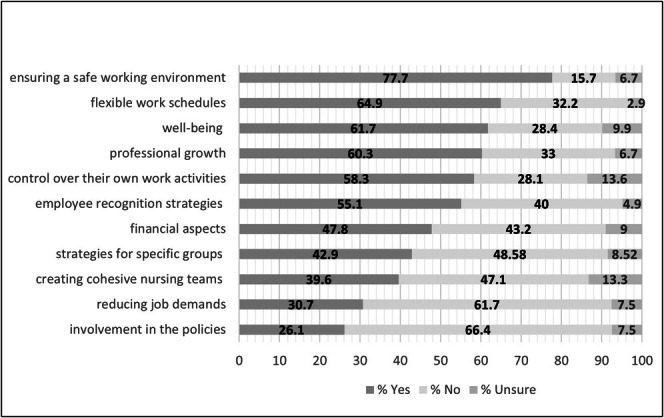
Percentage of responses to experience of types of retention strategies.

**FIGURE 2:**
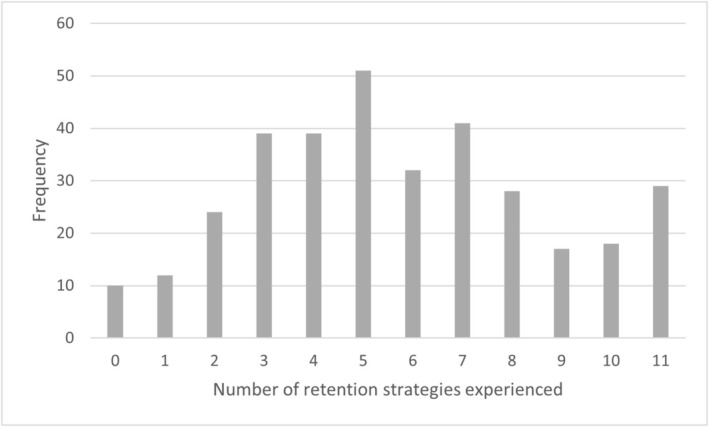
Frequency of number of strategies experienced by each participant.

The most frequently reported strategy experienced was attention to a safe working environment, by 75% of participants. Over 50% of participants reported experiencing strategies related to flexible work schedules (65%), well‐being (64%), professional development opportunities (60%), control over work activities (58%) and employee recognition strategies (55%) (Figure 1). The least frequently reported strategies experienced were involvement in service policymaking (26%), reducing job demands (31%); creating cohesive nursing teams (40%); strategies for specific groups (43%) and attention to financial aspects (48%). Most participants reported experiencing between four and seven types of retention strategies, while small numbers reported experience with very few (0–1) or very high (10–11) numbers of strategies (Figure 2).

### 4.3 Variation in Experience of Retention Strategies by Job and Work Characteristics

Those working in affluent areas were significantly more likely to report experience of the following types of retention strategies than those working in other types of socio‐economic areas: flexible working; attention to financial benefits; control over work activities; workload reduction strategies; involvement in service decision making; wellbeing strategies; and strategies targeting specific groups such as new to community nursing (Table 2). Those participants paid at higher NHS pay bands were significantly more likely to report strategies for professional growth and involvement in service policies and decision‐making than those paid at lower NHS pay bands (Table 2). Additionally, participants working in towns were significantly more likely to experience cohesive nurse team strategies, and those who work in a community interest company were significantly more likely to report employee recognition strategies (Table 2). Other job and work characteristics did not show statistically significant effects (*p* > 0.05) on reported experience of retention strategies (Table 2).

**TABLE 2:** Summary results of the Multinominal Logistic Regression analyses.

Predictor variables	Categories	Strategies	Professional growth opportunities (Likelihood‐Ratio Index = 0.0047**, Pseudo *R* ^2^ = 0.0702)		Flexible working strategies (Likelihood‐Ratio Index = 0.0307*, Pseudo *R* ^2^ = 0.0665)		Control over Work activities (Likelihood‐Ratio Index: 0.0046**, Pseudo *R* ^2^: 0.0636)		Financial benefits (Likelihood‐Ratio Index: 0.0085**, Pseudo *R* ^2^: 0.0612)		Cohesive nurse teams (Likelihood‐Ratio Index: 0.0085**, Pseudo *R* ^2^: 0.0510)		Employee recognition strategies (Likelihood‐Ratio Index: 0.0139*, Pseudo *R* ^2^: 0.0642)		Reducing workload strategies (Likelihood‐Ratio Index: 0.0139*, Pseudo *R* ^2^: 0.0642)		Safe Work environment strategies (Likelihood‐Ratio Index: 0.1417, Pseudo *R* ^2^: 0.0595)		Active involvement in policies (Likelihood‐Ratio Index: 0.0015**, Pseudo *R* ^2^: 0.0806)		Well‐being strategies (Likelihood‐Ratio Index: 0.3264, Pseudo *R* ^2^: 0.0371)		Strategies targeting special groups (Likelihood‐Ratio Index: 0.1742, Pseudo *R* ^2^: 0.0415)	
		Outcome	Coef	*p*	Coef	*p*	Coef	*p*	Coef	*p*	Coef	*p*	Coef	*p*	Coef	*p*	Coef	*p*	Coef	*p*	Coef	*p*	Coef	*p*
NHS pay band	4–8	Maybe	−0.398	0.100	−0.239	0.455	−0.162	0.339	0.024	0.900	−0.165	0.272	0.376	0.110	−0.099	0.604	−0.132	0.563	−0.10	0.59	−0.33	0.076	−0.06	0.748
		Yes	0.274*	0.012	0.133	0.216	0.163	0.143	0.189	0.065	0.061	0.568	0.132	0.199	0.113	0.311	0.012	0.927	0.31*	0.01	0.03	0.760	0.17	0.101
Socio‐economic segments	Mixed with pockets of affluence and deprivation	Maybe	−0.462	0.517	0.982	0.400	1.748	0.005**	1.887	0.077	−0.461	0.305	0.657	0.443	0.815	0.244	1.173	0.120	0.07	0.91	0.19	0.723	0.12	0.852
		Yes	−0.770*	0.030	0.077	0.814	0.628	0.070	0.336	0.288	−0.005	0.988	−0.100	0.754	−0.022	0.950	0.650	0.091	0.03	0.93	0.44	0.197	−0.21	0.505
	Mainly socio‐economically affluent	Maybe	−1.850	0.156	2.968	0.060	0.624	0.548	−12.35	0.984	−1.785	0.121	−14.92	0.993	0.324	0.758	0.727	0.607	−0.31	0.76	−0.81	0.507	−0.69	0.582
		Yes	−0.452	0.405	1.924**	0.002	1.253	0.027*	1.312**	0.008	0.718	0.157	0.848	0.108	1.198*	0.020	1.323	0.074	1.27*	0.02	1.21*	0.037	0.98*	0.050
Organisations	NHS community service trust	Maybe	−0.657	0.336	−0.065	0.956	0.024	0.969	0.528	0.458	−0.629	0.094	0.022	0.980	1.129	0.294	14.54	0.986	−0.09	0.89	0.45	0.529	−0.57	0.351
		Yes	−0.230	0.551	−0.640	0.118	−0.256	0.524	−0.096	0.791	−0.206	0.803	−0.584	0.119	−1.018**	0.007	−0.399	0.398	−1.17**	0.003	−0.31	0.434	−0.30	0.411
	Community interest company (or other type of social enterprise business)	Maybe	−0.998	0.414	−1.473	0.991	−1441	0.980	0.527	0.572	0.527	0.572	−1.157	0.377	0.652	0.608	14.23	0.986	−0.11	0.91	0.12	0.900	−0.68	0.455
		Yes	0.110	0.837	−0.788	0.144	−0.639	0.222	0.024	0.963	−0.426	0.409	−1.243*	0.016	−1.726**	0.006	0.355	0.633	−0.57	0.28	−0.71	0.185	−0.34	0.509
	NHS acute and community service trust	Maybe	−0.111	0.884	−0.094	0.941	0.866	0.178	0.366	0.626	0.342	0.568	−0.176	0.850	1.337	0.224	15.17	0.986	−0.20	0.79	0.57	0.462	0.02	0.969
		Yes	0.145	0.725	−0.514	0.234	0.158	0.716	−0.162	0.673	−0.640	0.109	−0.434	0.272	−0.475	0.218	0.108	0.838	−0.95*	0.02	0.00	0.999	0.00	0.992
Geographic settings	Town	Maybe	−1.579	0.053	0.261	0.838	0.601	0.378	−1.946*	0.029	−0.952	0.083	−1.784	0.164	−0.757	0.324	0.421	0.654	−1.33	0.09	−0.41	0.533	−1.00	0.186
		Yes	−0.567	0.171	−0.454	0.271	−0.002	0.997	−0.679	0.086	−0.926*	0.026	−0.616	0.112	−0.545	0.200	0.012	0.980	−0.84	0.05	−0.56	0.202	−0.53	0.174
	Inner city	Maybe	−0.564	0.577	0.471	0.770	0.772	0.427	−0.883	0.463	−1.100	0.221	1.293	0.226	1.093	0.226	1.696	0.197	−0.11	0.91	0.00	0.997	−0.04	0.966
		Yes	−0.723	0.225	−1.140	0.050	−0.112	0.861	−0.810	0.159	−0.466	0.425	−0.358	0.544	0.518	0.394	0.885	0.305	−0.68	0.29	−0.39	0.536	−0.38	0.504
	Rural	Maybe	0.185	0.805	−15.02	0.985	−0.302	0.689	−0.197	0.781	−0.663	0.327	0.890	0.356	0.362	0.623	−0.422	0.718	0.29	0.68	−0.49	0.530	−0.59	0.450
		Yes	0.381	0.441	−1.136*	0.014	−0.468	0.340	−0.756	0.102	0.148	0.746	0.034	0.940	−0.029	0.951	0.091	0.872	−0.11	0.81	−0.43	0.392	−0.78	0.084
	Mixed urban/rural	Maybe	−1.346	0.068	0.277	0.530	−0.274	0.670	−0.327	0.586	−0.195	0.700	−0.067	0.940	−0.453	0.503	0.930	0.306	−0.51	0.42	−0.71	0.275	−0.15	0.803
		Yes	−0.014	0.972	−0.253	0.452	−0.751	0.067	−0.633	0.104	−0.375	0.341	−0.362	0.333	−0.696	0.093	0.300	0.542	−1.01*	0.02	−0.70	0.098	−0.78*	0.042

* *p* < 0.05.

** *p* < 0.01.

### 4.4 Views on Effective Strategies

One hundred and thirty‐four free text comments were made on the perceived effectiveness. Of these, 69 were comments on the perceived effectiveness of strategies and 57 were negative comments in terms of effectiveness. Eight comments only gave more detail of how they had experienced the strategy but did not make any comment on effectiveness.

[we were] given £20 per head per team for a team building activity of our choice. It was really nice and we all got to have a nice meal together outside of the christmas meal and work desks. (ID 906)

There was only one comment left in response to the strategy of control over ones work to query why it had been added to a survey aimed at nurses in district nursing services as they stated, ‘Autonomy is the nature of the job in community’ (ID 101).

The strategies with the most comments in support as effective were those that: targeted sub‐groups in the nursing workforce (*n* = 20); addressed finance and salary (*n* = 11); provided flexibility in work schedules (*n* = 9); addressed the nurses' wellbeing (*n* = 8); addressed occupational health and safety (*n* = 6); addressed professional development (*n* = 6); addressed workload (*n* = 5); and staff recognition schemes (*n* = 1). We report each of these strategies in turn.

Participants provided the most comments on the effectiveness of strategies and programmes targeted at four groups: newly qualified nurses, nurses new to‐district nursing, retirement‐age nurses and internationally educated nurses. They often provided quantifiable evidence of effect.

Community newly qualified nurses now have their own preceptorship programme as the “acute” [hospital based] programme was not suitable for them. In community the induction period is of at least 8 weeks where they are supernumerary where in the acute sector it is just 2 weeks. Last September's intake of 16 newly qualified nurses has led to a retention of 15. (ID820)

The two comments which reported strategies targeted at subgroups as ineffective explained that these the strategy had been for hospital‐based nurses rather than specific to the district nursing service.

The next most frequently reported successful retention strategy was that which paid attention to pay and finance. Some participants reported that paying nurses the specialist district qualifications on NHS pay band seven, in contrast to other local employers paying them at a lower NHS pay band, had helped retain that group in their organisation. Some participants reported their organisation was increasing the financial reimbursements for fuel and use of the staff member's own car in recognition of high rates of inflation. Another strategy reported to be effective was the provision of pool cars (i.e., organisation owned cars that could be borrowed) rather than the nurses using their own cars.

We have the following—pool car (limited number but support those hitting the mileage cap but anyone can book to use), claim mileage/expenses, salary sacrifice car scheme and childcare scheme available. These all help keep staff. (ID 301)

The four negative comments about financial strategies all concerned the implementation in their organisation in which lower financial levels were offered compared to other organisations.

There have been issues with regards to fuel expenses payments within our trust being at a far lower level than that offered by others locally. I think this has impacted upon retention especially due to the cost of living increases many people are experiencing. (ID 315)

The fourth most frequently reported effective strategy was the introduction of a flexible work schedule and self‐rostering.

Allowing staff to submit flexible working requests to support work life balance, self‐rostering has had a positive impact on retaining staff. (ID 405)

Several of these comments also included caveats such as the importance of the policies being seen to be applied fairly and the challenge of flexible working requests often presented to the middle managers responsible for staffing a wider area or several teams. This strategy received no negative comments on effectiveness.

The fourth most frequently commented on as effective retention strategies were those that paid attention to manageable workloads. Some of these were very specific; for example, limiting the number of home visits any nurse undertook in a shift to ensure the accompanying patient administration was completed within work time. Others reported multiple strategies:

Safer staffing review with recommendations aligned to QNI [Queen's Nursing Institute] workforce standards has helped support more manageable caseload sizes keeping staff well in work. Streamlined paper records has helped reduce stress marginally. (ID 005)

Strategies to support the wellbeing of nurses received the next most frequent positive comments. These included organisational level provision of counselling services, mindfulness courses and apps, Schartz rounds, professional nurse advocate services, as well as activities within individual teams.

wellbeing wednesday in the office for cakes etc./direct access to wellness team. All of these makes people feel supported and therefore want to stay in this working environment. (ID 019)

Some reported that many of the wellness strategies had been present during the pandemic but had since been stopped or were only available in their NHS trusts to onsite hospital nurses. While others queried these services' impact on actual retention.

Counselling has been useful. I don't think it directly affects retention of nurses. (ID 012)

Strategies for occupational health and safety received the next highest number of comments as effective, particularly in relation to safety and lone working. Examples given were the provision of personal alarms, work mobile phones, working in twos in the evening shifts. However, there were 13 comments arguing that these strategies were not effective in the retention of nurses. Some comments implied this was expected policies for NHS organisations and therefore irrelevant to the nurses' decision‐making in leaving a post. Others implied that the way the lone working polices were implemented resulted in nurses feeling unsafe and leaving.

Staff have often requested to work in two's on twilight shift due to safety and this would improve retention, however unless the area is deemed as unsafe, this is declined. (ID506)

Planned opportunities for professional growth and career progression in the district nursing service received the next most frequent positive comment on effectiveness in retention, particularly linked to career progression.

Growing our own staff is effective in retention, taking staff through apprenticeships from band 3 [health care assistant level] up to ACP [advanced clinical practitioner] level. (ID705)

Strategies to reducing workload and burden on nurses received five comments of effectiveness for retention and some detail as to what they were such as; paper lite record keeping systems, using tools to identify and act on issues in staffing capacity, limiting numbers of visits per nurse per shift.

limit visits per day to ensure all admin is completed and not delayed until the nurses' own time. (ID 004)

However, four comments were made on the absence of such strategies, such as this; ‘Workload is only ever added to’. (1D 007).

Only one comment considered employee recognition strategies as helpful in retention. In contrast, the largest group of comments (*n* = 23) refuted employee recognition strategies as they had experienced them as effective in retaining staff.

Nurse of the month was commenced but this created upset within the team as every member works hard and of a high standard, therefore, why should all the team not be acknowledged. (ID 221)

## 5 Discussion

This national survey demonstrated that not all registered nurses and nursing associates in district nursing services experienced the full range of retention strategies advised for nurses by international authorities (World Health Organization 2016; Buchan et al. 2022). To our knowledge, this is the first study to provide such evidence for district nursing services, where nurse turnover rates in several countries are known to be higher than those for hospital employed nurses. While a study of US home care nurses, conducted 20 years ago, suggested that their employing agencies did not implement all retention strategies, the researchers did not have specific evidence as such (Ellenbecker et al. 2007).

In this study, the most frequently reported strategies experienced by nurses were that of occupational health and safety strategies, and then flexible work schedules. However, over 30% of participants reported they had not experienced flexible work schedules, a strategy with significant evidence in support of its retention capabilities (World Health Organization 2016; Buchan et al. 2022), including from the free text testimony of participants in this survey. The retention strategies experienced reported by the lowest number of participants were those that: involved nurses in the policy decision‐making of their service, and those that made the workload manageable. While commentary from American research with home care nurses suggested that involvement in policy decision‐making was important to retention (Ellenbecker 2004), free text comments in this survey questioned whether such strategies had a direct effect on the retention of nurses. This issue requires further investigation, perhaps in a study in multiple countries, and in the context of models such as Buurtzorg derived self‐managing district nursing teams (Hegedüs et al. 2022).

While free text commentary reported strategies to create manageable workloads, such as implementing guidance on safe patient caseloads and limiting the number of home visits per shift, these were not strategies experienced by most participants. Funding mechanisms for home visiting nursing services differ between countries. In some, such as the Netherlands and the US, payment is through insurance systems in which payment is allocated per individual patient. In England, the NHS mainly funds district nursing by a block contract system, that is, a fixed amount of money for a general population rather than by activity levels (NHS Providers 2018). These results raise questions as to the effects of the overall contracting system on organisational retention strategies. One could speculate as to whether different contracting mechanisms could improve the ability of individual organisations to have strategies for manageable workloads in district nursing, with concomitant effects on nurse retention. This requires further investigation in a study in multiple countries with different funding mechanisms.

The survey provided evidence for the first time that subgroups within district nursing services had different experiences of retention strategies when adjusting for other factors that may also be influential. Those nurses and nursing associates on lower pay bands were statistically significantly less likely to report experience of strategies for professional growth and involvement in service policy decision‐making than those paid on higher bands. This has not been reported before; most studies of nurses' experiences of continuing professional development have been undertaken with those employed in hospitals and have not considered workplace seniority but rather factors such as age and gender (Vázquez‐Calatayud et al. 2021). This finding requires further investigation as to whether this differential experience exists in other countries. Possible explanations for this finding could include the tight financial environments, or perhaps that the hierarchical nature of nurse teams is then reflected in resource allocation to support professional growth. Either way, this could be an area for further investigation and a key self‐assessment point for district nursing service organisations concerned about retention levels in the groups that make up most of its staff.

We report for the first time that those employed in organisations providing community and mental health services were statistically significantly more likely to report experiencing strategies to manage or reduce workloads than those employed in other types of organisations. A possible explanation might be that senior staff in these types of organisations had experience of providing community services to people with mental health problems and therefore a different viewpoint as to manageable workloads. This would require further investigation and perhaps international comparisons where home‐visiting nurses are employed by hospitals or by stand‐alone home care agencies.

Those participants working in rural areas were statistically significantly more likely to report that there were strategies to support cohesive teams than those working in other types of areas. A possible explanation might be that working alone over large areas and periods of time was more obvious than in other areas and thus greater attention was paid to creating team opportunities. Likewise, those in rural areas reported greater involvement in service policymaking than those in other areas. Perhaps this type of activity was also part of the team‐building activities. This requires further investigation and testing, including in countries with significant remote and rural areas.

Strikingly, we report for the first time that participants working in affluent areas were statistically significantly more likely to report experiencing the following strategies than those working in socio‐economically deprived areas: attention to pay and financial aspects; flexibility in work schedules; control over work activities; strategies for manageable workloads; strategies for health and wellbeing; involvement in service policy and decision‐making; and strategies for specific groups such as newly qualified nurses. This requires further investigation in studies in other countries to establish whether this is an issue specific to one country or is found elsewhere. Possible explanations in England are that more affluent areas have historically received greater government funding than impoverished areas (Barr and Taylor‐Robinson 2014) and this has continued through the block contract system, allowing managers greater flexibility in resourcing strategies to support retention. This requires further investigation.

### 5.1 Strengths and Limitations

This study offers significant strengths that contribute to the robustness and generalizability of its findings. One of the primary strengths is the relatively large sample size, consisting of 345 nurses and nursing associates. Additionally, participants are drawn from a variety of geographical locations, organisations and socio‐economic backgrounds, which further bolsters the study's representativeness. However, a response rate cannot be calculated as the membership numbers in the professional on‐line networks were unknown.

However, despite these strengths, there are limitations that need to be acknowledged. The study did not gather information on participant variables such as age, ethnicity and years of experience. These factors could have enhanced the depth of the analysis. For example, ethnicity could influence cultural perceptions and experiences within the workplace, while years of experience might affect how nurses respond to different retention strategies, with more experienced nurses potentially valuing different aspects of their work environment compared to those who are newer to the profession (Murray et al. 2016). These individual factors were not controlled for in the regression analysis, and this could also be seen as a limitation; however, the aim of the study was to investigate organisational characteristics rather than individual characteristics. A further limitation was that the survey used self‐report rather than an objective measure for factors such as the affluence or otherwise of the area that the nurse worked in. The decision to use self‐report rather than ask the nurse to find out an objective measure, such as the area score of indices of multiple deprivation, was made to ensure the nurse completed the question without having a separate task, which the study advisory group considered would not have been undertaken by busy nurses.

### 5.2 Recommendations for Further Research

The research was undertaken in the context of one country with a tax funded national health service and multiple types of provider organisations. The evidence reported here may not be the same for other countries with differently organised home visiting services. Further studies repeating this investigation in multiple countries would identify the evidence that is applicable across systems or specific to services organised in a similar way.

### 5.3 Implications for Policy and Practice

The findings suggest that clinical leaders of district nursing services, experiencing high turnover rates, should consider reviewing whether the full range of advised retention strategies is available and implemented in their organisations. We suggest that nursing leaders at team and more senior levels review the extent to which the retention strategies are experienced equitably throughout the nursing team or whether they are inequitably experienced by virtue of seniority in pay, the work base and area, or other individual characteristics such as age, gender and ethnicity.

## 6 Conclusions

Retention strategies are created and enacted by employing organisations, district nursing services and district nursing teams, but within the context of a wider health care and labour market system. This study aimed to investigate effective retention strategies for nurses and nursing associates in district nursing services. International guidance advises a suite of strategies generic for any type of nurse. This national survey found that views on effective strategies were mixed, but attention to financial aspects, manageable workloads, flexibility in work scheduling plus tailored induction/support for those new into district nursing were given the most testimony as effective. We found that not all nurses and nursing associates experienced the range of advised strategies, and some, such as those on lower pay bands and working in socio‐economically deprived areas, are significantly less likely to experience them. Our findings raise several research questions for subsequent investigation across health systems.

## Author Contributions

**Erkan Alkan:** investigation, data curation; formal analysis; project administration writing – original draft; writing – review and editing draft. **Vari M. Drennan:** conceptualisation, funding acquisition, methodology, investigation, analysis, supervision; writing – review and editing. **Claire Thurgate:** conceptualisation, funding acquisition, methodology; writing – review and editing. Mary Halter: conceptualisation, funding acquisition, methodology; writing – review and editing. **Lihua Wu:** conceptualisation, funding acquisition, methodology; writing – review and editing. **Chao Wang:** supervision; writing – review and editing.

## Ethics Statement

The study (application 3338) was reviewed and approved by the Kingston University Research Ethics Committee.

## Conflicts of Interest

The authors declare no conflicts of interest.

## Supporting information


**Data S1:** Text of the questions included in the online survey.

## Data Availability

The data that support the findings of this study are available from the corresponding author upon reasonable request.
