# Supplementary material for: Experience and Perceptions of Retention Strategies in District Nursing Services: A Web‐Based Mixed Methods Cross‐Sectional Survey
Source: J Adv Nurs. 2025 Aug 1;82(4):3767–78. doi: 10.1111/jan.70108 (PMC12994618; doi:10.1111/jan.70108)
Supplement: Supplementary file 1 — Data S1: Text of the questions included in the online survey. [file JAN-82-3767-s001.docx]

**Experience and perceptions of retention strategies in district nursing services: a web-based cross-sectional survey.**

**Supplementary file 1 Text of the questions included in the online survey**

**Retaining community nurses: the survey**

Section 1: About your job

1. Please add your job title below

FREE TEXT

1. Please select your current NHS Agenda for Pay Band

DROP DOWN LIST BANDS 1 TO 8C, OTHER AND SPACE

**Section 2: Your views on retention strategies**

Team leaders, managers and employers may use a range of strategies to help retain community nurses and reduce turnover. We have identified a number from our literature review.

Please respond on the drop-down lists to any of the following types of strategies and if you have experienced them.

|  | Yes | No | Unsure |
| --- | --- | --- | --- |
| Some organisations promote **professional growth**, such as offering nurses opportunities for advancement through internal promotion or educational pathways. Have you experienced this strategy? |  |  |  |

Please briefly describe the types of strategies you've encountered (e.g., professional development plans for every nurse, training opportunities) and whether you found them effective in retaining you or your colleagues within your jobs or the organisation.

FREE TEXT

|  | Yes | No | Unsure |
| --- | --- | --- | --- |
| Some organisations use **flexible work schedules,** including options such as individual control over hours and rota arrangements, including various shift patterns like short shifts, long days, twilight shifts, and overnight shifts, with a focus on retention strategies. Have you experienced this for community nurses? |  |  |  |

Please briefly describe the type of flexible working strategies you have experienced e.g. self-rostering, and whether you considered this was effective in retaining you or your colleagues in community nursing jobs.

FREE TEXT

|  | Yes | No | Unsure |
| --- | --- | --- | --- |
| Some organisations promote the strategy of nurses having **control over their own work activities**, e.g. nurses at the team level have the authority and independence to make decisions in the care they provide, and the freedom to plan and organize their work. Have you experienced these types of strategies? |  |  |  |

Please briefly describe the type of strategy promoting control over work activities that you have experienced e.g. self-managing teams , and your views on their effectiveness or otherwise in helping retain community nurses.

FREE TEXT

| **Category** | **Yes** | **No** | **Maybe** |
| --- | --- | --- | --- |
| Some organisations have paid**attention to the financial aspects of the jobs and access to other benefits.**For example, ensuring salaries/grades are the same or better to neighbouring organisations, providing access to other benefits such as staff childcare, provision of transport (e.g. staff pool cars, e-bikes), facilitating the process of claiming expenses, such as petrol money reimbursement, to enhance convenience and efficiency for employees**.**Have you experienced any of these types of strategies? |  |  |  |

Please briefly describe the type of financial or other benefits you have experienced and whether you considered they were effective in retaining community nurses?

FREE TEXT

|  | Yes | No | Maybe |
| --- | --- | --- | --- |
| Some organisations have focused on **creating cohesive nursing teams**e.g. teams of nurses who work well together and support each other. Have you experienced any such strategies in your employment as a community nurse? |  |  |  |

Please briefly describe the type of strategy you have experienced in creating cohesive nursing teams e.g. team building sessions, and your views as to whether they were effective in retaining community nurses?

FREE TEXT

|  | Yes | No | Maybe |
| --- | --- | --- | --- |
| Some organisations use **employee recognition strategies**e.g. nurse of the month awards for good or exceptional performance. Have you experienced any such strategies? |  |  |  |

Please briefly describing the type of strategy e.g. a scheme to nominate colleagues for awards (which may be financial or non-financial) and whether you considered it was effective or otherwise in retaining community nurses.

FREE TEXT

|  | Yes | No | Maybe |
| --- | --- | --- | --- |
| Some organisations have paid attention to**reducing job demands**e.g. reduce nurses’ stress and workload, possibly including provisions for remote working. Have you experienced any strategies that have tried to address workload and burden of work? |  |  |  |
|  |  |  |  |

Please briefly describe the type of strategy for reducing workload e.g., a safe staffing policy to limit case load size or schemes to reduce paperwork, and whether you thought it was effective in retaining community nurses?

FREE TEXT

|  | Yes | No | Maybe |
| --- | --- | --- | --- |
| Some organisations have paid attention to **ensuring a safe working environment** e.g. provision for working in pairs, provision of mobile phones, or offering personal protection devices like Sky Guard devices?  Have you experienced strategies which have paid attention to safe working environments? |  |  |  |

Please briefly describe the type of strategy you have experienced e.g., policies on lone working and your view as to whether it was effective in retaining community nurses.

FREE TEXT

|  | Yes | No | Maybe |
| --- | --- | --- | --- |
| Some organisations have paid attention to ensuring nurses**’ active involvement in the policies, review, and decision-making about the whole home visiting nursing service**e.g., a nurses’ council.  Have you experienced any such strategies? |  |  |  |

Please briefly describe the type of strategy you have experienced e.g., a community nursing practice and policy committee, and your views on their effectiveness or otherwise in retaining community nurses.

FREE TEXT

|  | Yes | No | Unsure |
| --- | --- | --- | --- |
| Some organisations have strategies focused on community nurses' **well-being**e.g. access to classes for improving physical and/or mental health, implementation of wellbeing conversations. |  |  |  |

Briefly describe any wellbeing strategy you have experienced e.g. mindfulness sessions, access to counselling services and your views as to how effective they were in retaining community nurses.

FREE TEXT

|  | Yes | No | Maybe |
| --- | --- | --- | --- |
| Some organisations have retention strategies for **specific groups in community nursing** e.g. newly qualified nurses, new to community services, internationally educated nurses, nurses close to retirement. Have you experienced or observed such strategies ? |  |  |  |

Briefly describe any strategies for specific groups and your view as to whether they were successful in retaining the target group in community nursing.

FREE TEXT

**Section 3. Any other views on successful retention strategies?**

Please add any comments you would like to add about successful (or otherwise) retention strategies for home visiting community nurses that you have not mentioned above.

**FREE TEXT BOX**

**Section 4: About your work area**

**What type of area do you work in? Please choose from the drop-down list.**

Mainly Rural

Town

Inner City

Outer city and/or suburbs

Mixed Urban and Rural

Other (please describe)

**How would you describe your work area? Please choose from the drop-down list:**

Mainly socio-economically affluent

Mainly socio-economically deprived

Mixed with pockets of affluence and deprivation

Other (please describe)

**Which region do you work in? choose from the drop-down list?**

East of England

London

Midlands

North East and Yorkshire

North West

South East

South West

**What type of organisation are you employed in? choose from drop down list?**

NHS Acute and Community Service Trust

NHS Community Trust

NHS Community and mental health trust

Community Interest Company (or other type of social enterprise business)

Limited Company (or other type of for-profit business)

Other (please state)

**Thank you for your time. This is the end of the survey.**
